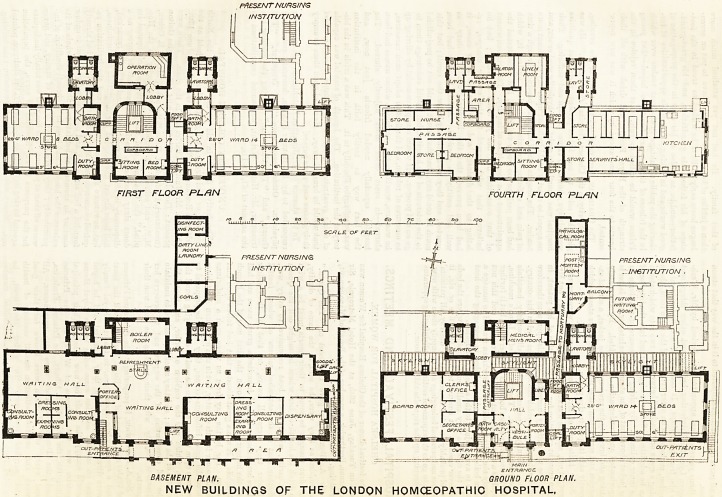# Practical Points

**Published:** 1894-06-30

**Authors:** 


### PRACTICAL POINTS.

[Questions are invited for this column on any point in the administration of hospitals and asylums which may prove of difficulty or interest to our readers. All communications should be marked "Practical Points," addressed to the Editor of The Hospital, 428, Strand, W.C.]

*Disinfecting Chamber.—A Correspondent writes: What is the most satisfactory apparatus of this description to be used for a small country hospital where the fever block contains five or six beds?*

The effectual destruction of the living organisms which, under various names, are the active agents in propagating infectious diseases is one of the most difficult problems with which the managers of hospitals have to cope. Formerly it was thought sufficient to fumigate with burning sulphur or to subject clothes and other articles which required to be disinfected to heat generated either by gas or by coal. The researches of Dr. Parsons and of others following him have, however, proved conclusively that, while sulphurous acid is effective in some cases, and a moderate amount of dry heat in others, the only process of destruction upon which any reliance can be placed in dealing with the specific germs of infectious disease is steam at high pressure. Dry heat, besides being destructive to many kinds of clothes, and especially so if raised beyond a certain temperature, has little or no penetrative power, and does not therefore reach

FIRST FLOOR PLAN

FOURTH FLOOR PLAN

BASEMENT PLAN.

GROUND FLOOR PLAN.

### NEW BUILDINGS OF THE LONDON HOMŒOPATHIC HOSPITAL.

to all parts of the things subjected to it in the way that steam does.

Some years ago an apparatus was devised by Mr. Washington Lyon for disinfecting bedding and other things sent to him from large ships It consists of a cylinder, oval in section, enclosed in an outer cylinder of the same form, there being a space or "jacket" between the two into which steam can be admitted. Steam is also admitted into the interior of the inner cylinder. By raising the pressure of the steam in the jacket above that of the steam in the interior, the latter is superheated and condensation is avoided. By alternately raising and lowering the pressure in the jacket, a sort of expansion and contraction takes place in the inner steam, which greatly aids its penetrative power.

Some imitations of Mr. Lyon's apparatus were recently brought out, and one was somewhat widely adopted; but Mr. Lyon brought an action for the protection of his patent, in which he was entirely successful, and his apparatus therefore remains the only one which can lega lly be used in this country.

The difficulty experienced in small country hospitals is the question of cost. The first cost of a Lyon's machine, with its necessary steam boiler, is considerable; but it further requires a certain amount of skill in working; and this not merely the ordinary skill of a workman trained to manage a steam boiler, but care and knowledge in managing the heat so as to avoid injury to the articles disinfected.

In the case of a very small country hospital where the beds for fever cases do not amount to more than five or six, it would probably be more economical to destroy all patients' clothes and personal belongings and to supply them with new, than to go to the expense of setting up a disinfecting apparatus and keeping up the requisite skilled labour.

## Figures and Tables

**Figure f1:**